# How effective are perches in promoting bird-mediated seed dispersal for natural forest regeneration? A systematic review protocol

**DOI:** 10.1186/s13750-023-00308-z

**Published:** 2023-08-03

**Authors:** Jelaine Lim Gan, Matthew James Grainger, Mark David Foster Shirley, Marion Pfeifer

**Affiliations:** 1 School of Natural and Environmental Sciences, Newcastle University, Newcastle upon Tyne, UK; 2 Norwegian Institute for Nature Research, Torgarden, Postbox 5685, 7485 Trondheim, Norway; 3 Institute of Biology, College of Science, University of the Philippines, Diliman, Quezon City, Philippines

**Keywords:** Reforestation, Regrowth, Avian, Frugivores, Artificial perch

## Abstract

**Background:**

Forest landscape restoration (FLR), often through tree planting, is one of the priorities in many global and national initiatives for carbon offsetting as part of climate change mitigation and biodiversity conservation. However, active efforts to meet FLR objectives entail substantial costs for the procurement of planting stocks and require an experienced workforce for planting and nurturing tree seedlings. Alternatively, restoration projects can be more cost-effective and potentially may have greater biodiversity gain through assisting and accelerating natural forest regeneration. The use of perches is one of the strategies under Assisted Natural Regeneration (ANR) and is used to attract avian seed dispersers to degraded habitats for increased tree seed supply and seedling establishment. This systematic review and potential meta-analysis aim to determine the effectiveness of artificial and natural perches in promoting natural forest regeneration. Specifically, we will evaluate their effectiveness in driving seed richness, seed density, seedling richness, and seedling density. The results will synthesize available evidence on the topic, identify knowledge gaps we need filling to upscale the strategy, and inform their use in concert with other ANR strategies.

**Methods:**

The search strategy was informed through a literature scan and discussions with stakeholders and experts. A total of eight databases, which include an organizational library and a web-based search engine, will be searched using the refined search string in English. The search string was formed using keywords corresponding to the PICO structure of the research question, and its comprehensiveness was evaluated using 10 benchmark articles. The search results will be screened by the review team (composed of a primary and at least two secondary reviewers) using the set eligibility criteria at the title and abstract level, followed by the full-text screening. The screened studies will then undergo critical appraisal using the assessment criteria based on risk of bias and methods. Data from the accepted studies will be extracted to the standard data sheet for meta-analysis. Effect size (Hedges’ g) will be computed to determine whether perches are effective in increasing seed dispersal and seedling establishment in degraded sites. The effect of potential modifiers relating to the landscape will be explored via mixed models.

**Supplementary Information:**

The online version contains supplementary material available at 10.1186/s13750-023-00308-z.

## Background

Tropical forests are threatened ecosystems that support high levels of biodiversity and crucial ecosystem services [1–3]. They play a significant role in the carbon cycle, acting as sinks as well as sources [4, 5], and in regulating water and energy cycles [6]. Globally, they are decreasing at a rapid pace owing to human activities such as logging [7, 8], agricultural expansion [9], and urbanization [10]. Tropical forests account for more than 90% of the global deforestation, with 157 Mha of forest area lost from 2000 to 2018 [11]. If left unabated, such rapid forest loss is likely to result into a protracted mass extinction of forest dependent species [2] and impaired ecosystem functioning [12].

International and national institutions alike have recognized the urgency of protecting remaining forests and restoring degraded lands. Net zero forest loss is a key target in international conventions and declarations (e.g., Bonn Challenge, United Nations Sustainable Development Goals, COP26 climate summit) [13]. Reflecting the critical need to reverse and mitigate deforestation to meet commitments, forest landscape restoration is gaining more attention in research and practice at national and local scales [13, 14]. However, widely used reforestation activities such as monoculture or mixed planting of exotic and/or commercially valuable species in degraded habitats [15, 16] produced ineffective ecological outcomes, failing to restore biodiversity and ecosystem functioning to levels observed in natural forests [15]. This was partly due to poor species—site matching and lack of understanding of the natural forest structure [17]. These activities were also costly, trying to ensure sourcing good planting stock and using extensive manpower for planting and nurturing [18].

Reforestation projects can be more cost-effective and potentially have greater biodiversity gains through accelerating natural forest recovery [14, 19]. Most forests are able to recover on its own, although the speed of recovery is dependent on intensity of past land use and time since restoration, among other factors [20, 21]. Assisted Natural Regeneration (ANR) is a reforestation strategy that accelerates forest succession by minimizing disturbances (i.e., fire, livestock) and competition (i.e., invasive species, weeds, vines) to the naturally regenerating trees [22–24]. ANR can also improve seed supply by promoting seed dispersal by animals such as birds, with bird dispersers shown to increase plant species richness in forest gaps and regenerating forests [25, 26].

Seed dispersal is a crucial process that has far-reaching effects on the plant population dynamics and ecological interactions [26]. This process of moving seeds away from the mother plant is often mediated by birds [27, 28], a service not exclusively provided by frugivores but also by omnivores. Generalist birds have been shown to improve forest regeneration through increasing abundance and diversity of the seed rain in degraded areas [25]. This bird-mediated process can also positively influence seed germination and seedling establishment, although results have been varied and species-specific [29–31].

However, birds in the forest are generally hesitant to visit degraded sites, because of increased risks from predation, harsher conditions, and lack of resources [32]. Several strategies have been tested to attract birds into the degraded areas as an ANR strategy. Methods include the use of artificial perches [33], applied nucleation or tree islands [34], planting of fruit trees [35, 36], and other natural structures used by birds as perches, sometimes in conjunction with supplemental water [37] and playback luring [38]. However, results from the habitat fragmentation literature suggest that the effectiveness of these perches may be affected by landscape and habitat factors. Birds may visit perches placed nearer the forest edge than those farther away [39]. The habitat around the perches also matters, as birds would prefer to move in areas structurally similar to their native habitat [40]. Vegetation structure, at perches and around them, will also alter water availability and soil moisture, known to affect the successful recruitment of seedlings from seeds [41].

To consolidate ANR strategies focused on attracting bird dispersers and thus to facilitate the upscaling of ANR across regions, an evidence synthesis is needed evaluating the effectiveness of various perching structures at degraded areas in promoting forest regeneration. Systematic reviews collate and synthesize critically appraised evidence to inform conservation practice, identifying interventions that are effective as well as knowledge gaps [42]. Contingent on data availability, a meta-analysis can also be performed by pooling data across studies to determine the overall effectiveness of the intervention on the outcome of interest and to explore potential modifiers or sources of heterogeneity. Guidetti et al. [33] conducted a meta-analysis on the use of artificial perches in 2015, but they neither included natural perches nor examined moderator effects of the landscape structure (e.g., matrix type, distance of perch to edge). This systematic review will summarize the importance of bird perching structures and discuss their effectiveness, advantages, and disadvantages, ultimately to guide interventions using perches in future ANR programs.

## Stakeholder/expert engagement

We have created an online stakeholder survey to seek insights and suggestions for our meta-analysis (Additional file 4). This was disseminated through email to experts in the field of forestry, ornithology, wildlife biology/ecology, restoration ecology, conservation science, and other relevant fields. To date, we have received nine responses from participants who were affiliated with different universities (based in UK, Thailand, Philippines) and environmental conservation organizations (BirdLife International, Instituto Claravis, Royal Society for the Protection of Birds). After explaining to them the overall research question and outlining the PICO (Table 1), they found the research question relevant and have suggested sub-topics for further exploration, including the dispersal distance of seeds from the forest. They have also suggested including ‘forest*’ (locator), ‘scrub*’ (intervention), and ‘regenerat*’ (outcome) as part of the search terms, which we have subsequently incorporated into the study design (Table 2).

**Table 1:** The PICO structure of the systematic review research question

Question key elements	
Population (P)	Degraded areas near a forest
Intervention (I)	Artificial perches (e.g., wooden posts, wires) Natural perches (e.g., single trees, shrubs)
Comparator (C)	Temporal: before and after intervention at the same site Spatial: with and without intervention at adjacent sites with the same expected seed source and ecoregion
Outcome (O)	Seed richness Seed density Seedling richness Seedling density
Moderator	Matrix type (e.g., agriculture, grassland, regenerating landscape) Distance of perch to forest edge Precipitation variation Bioclimatic region (tropical, subtropical, temperate, boreal) CEE Risk of Bias score (low, medium, high) Methods validity score (low, high)

**Table 2:** The proposed databases to search studies examining bird-mediated seed dispersal on perches

Source	Institutional subscription	Search fields
**Databases**		
Web of Science Core Collection	Newcastle University	Topic (includes title, abstract, author keywords, and keywords plus)
• Science Citation Index Expanded (SCI-EXPANDED) 1970–present		
• Social Sciences Citation Index (SSCI) 1970–present		
• Arts & Humanities Citation Index (AHCI) 1975–present		
• Conference Proceedings Citation Index—Science (CPCI-S) 1990–present		
• Conference Proceedings Citation Index—Social Science & Humanities (CPCI-SSH) 1990–present		
• Emerging Sources Citation Index (ESCI) 2015–present		
Zoological record	Newcastle University	Topic (includes title, book title, abstract, broad terms, descriptors data, super taxa, systematics, taxa notes)
SciELO Citation Index	Newcastle University	Topic (includes title, abstract, author keywords)
Scopus	Newcastle University	Article Title, Abstract, Keywords
CAB Abstracts	Newcastle University	WOK Free-Text index (English Item Title, Original Item Title, Source Abstract, CABICODE Names, Descriptors, Organism Descriptors, Geographic Location, Identifiers, Broad Terms)
ProQuest Natural Science Collection	Newcastle University	Title, Abstract, Keywords
**Websites**		
Conservation evidence	Open Access	NA Search studies using keyword “perch” and under category “birds”
**Web-based search engine**		
Google Scholar	Open Access	NA Search term: Bird AND perch AND “seed dispersal” (in English)

This list was compiled with the help of experts and stakeholders (see “Methods”)

## Objective of the review

This systematic review aims to provide a comprehensive summary on the common perches that can be used to attract bird dispersers for natural forest regeneration. It will assess the effectiveness of both artificial and natural perches in promoting seed dispersal and seedling establishment by examining evidence from literature and potentially through meta-analysis, data-permitting. We will also test if landscape and bioclimatic features are important covariates to observed effects. Seed germination as an outcome is not included, because it has been covered by Rogers et al. [30] in their meta-analysis.

### Primary question

How effective are natural and artificial perches in promoting bird-mediated seed dispersal and seedling establishment in degraded habitats? (Table 1).

### Secondary question

How do landscape and bioclimatic features alter the effectiveness of natural and artificial perches?

## Methods

This protocol follows the RepOrting standards for Systematic Review Syntheses (ROSES) for systematic review protocols (Additional file 5). The search strategy and search strings were discussed and improved upon with the help of an information specialist (Julia Robinson from Newcastle University Library).

### Search strategy

We will search for literature (e.g., articles, books, theses, institutional reports) through several databases (Table 2) and solicited calls for relevant papers. We will supplement this with searching of relevant references from review articles, including the references used by Guidetti et al. [33] in their review paper. No time limits will be applied in our search. A search update will be performed every year, if resources allow, until the review is published.

We expect to find most studies from the Web of Science Core collection and Scopus based on our initial scoping. We chose ten benchmark articles that provided evidence for different intervention types (i.e., artificial perches, shrubs, isolated trees, tree islands), to test the comprehensiveness of the proposed search string. All ten articles in the benchmark list were found in the former database, but one was missed in the Scopus search due to the lack of abstract. We included SciELO Citation Index to find regional studies from Latin America, Spain, Portugal, the Caribbean and South Africa, as well as ProQuest Natural Science Collection, CAB Abstracts, and Conservation Evidence for theses, reports, and conference proceedings that may not have been published and indexed. Web-based searches will also be done on Google Scholar engine as a supplementary source using terms in English. We will use main search terms from population, intervention, and outcome components, but only the first 200 results, sorted by relevance, will be examined. Lastly, to expand our search further, a public call for literature will be done through relevant mailing lists and social media (i.e., Facebook, Twitter).

### Search strings

Based on the elements of the question, we identified key terms that refer to the population, intervention, and outcome (Table 3). These were combined using ‘OR’ within each element and with ‘AND’ across to form the search string, such that an article will be returned if it referred to birds (and its synonyms), a type of perch, and a term about seed dispersal or seedling establishment.

**Table 3:** Search terms and search strings to be used for the meta-analysis

Elements	Search terms
Population	bird* avian aves disperse*
**Intervention**	
Artificial perches	perch* artificial perch* roost* wire* post*
Natural perches	palm* fruit* nucleation* nuclei tree isl* woodland isl* habitat isl* remnant tree* isolated tree* single tree* shrub* scrub*
Outcome	seed dispers* seed rain* seedling* regenerat*
Locator	forest* woodl*
Search string	(bird* OR avian OR aves OR disperse*) AND (palm* OR fruit* OR perch* OR “artificial perch*” OR roost* OR nucleation* OR nuclei OR “tree isl*” OR “woodland isl*” OR “habitat isl*” OR “remnant tree*” OR “isolated tree*” OR “single tree*” OR shrub* OR wire* OR post* OR scrub*) AND (“seed dispers*” OR “seed rain*” OR seedling* OR regenerat*) AND (forest* OR woodl*)

The final list of terms was generated through fine-tuning against ten benchmark articles and by working with stakeholder consultation

The search string was optimized for the Web of Science Core Collection by testing it against ten benchmark articles (Additional file 1). We included “disperse*” in the population term, because a paper in the benchmark list did not refer to birds specifically at the title, abstract, and keywords. For intervention, we added “roost*” to what Guidetti et al. [33] used for artificial perches and also included terms for natural perches. We used “forest*” and “woodl*” as the location term to exclude other habitats, such as grasslands or wetlands.

The final search string was then adjusted accordingly to the syntax of other databases. Due to resource constraints, we will restrict our search to publications in English, including literature with abstracts published in English but using other languages in the full text. We acknowledge that our search strategy may miss regional studies, but we tried to limit this bias by including references cited in review papers.

### Screening process

The screening of eligible studies will be done by the review team using a pre-defined inclusion/exclusion criterion, in accordance with Collaboration for Environmental Evidence (CEE) guidelines. We will create the ROSES flow diagram in ROSES flowchart R package [43] to report the number of papers excluded at every stage of screening—title and abstract, full text.

The inclusion review of the amassed library will be done in three parts. First, the library will be cleaned of duplicates based on DOI and title matches using the R package ‘revtools’ [44] and further screened using the de-duplication algorithm in the software Rayyan [45]. We have conducted a pilot run of the de-duplication process. From 11,801 articles collated from the databases (Additional file 1), the number of unique studies after DOI and title-matching was 5019. We then used Rayyan’s deduplication tool, and the program flagged 294 potential duplicates, which will be manually screened.

After the de-duplication process, we will conduct title and abstract filtering using the eligibility criteria below as guide, retaining uncertain ones for the next step. Lastly, we will conduct full-text filtering, primarily through reviewing the methods and results sections, as guided by the study inclusion criteria. The two-step filtering will be done in Rayyan to facilitate parallel screening, but we will manage the full-text articles in a separate reference management tool (e.g., Mendeley, EndNote). We will provide a supplementary list of articles that were excluded at this stage with reasons for exclusion.

All studies will undergo double-screening at both levels of filtering, following best practices guidelines [46]. The review team will consist of the primary reviewer and several secondary reviewers. The former will be leading and conducting most of the screening process, while the latter will conduct independent parallel screenings of subsets of the literature searches. The reviewers will evaluate each study and tag it as accept, reject, or unsure based on the eligibility criteria. The screening results, specifically the group disagreement rates, will be monitored weekly by the primary reviewer. If there is high disagreement rate (> 15%), the eligibility criteria will be re-evaluated and refined. Reconciliation meetings will be done after every 30% of the studies have been screened. Conflicting decisions will be discussed with the whole team until a consensus is reached. Reconciling decisions as the screening progresses, instead of tackling all conflicts at the end, reportedly saves time and improves the decision-making accuracy of the reviewers in the process [46]. This will be documented and made accessible as supplementary material. Meanwhile, studies tagged as unsure at title and abstract stage will be accepted to the next level of filtering and assessed at the full-text filtering stage. Those tagged as unsure at this final stage will be reviewed by the whole team.

### Eligibility criteria

We will review the collated studies obtained from the searches using the criteria set below (Additional file 2). This has been refined through pilot-testing on 20 articles selected from the initial search, intentionally chosen to represent articles that are clearly eligible, clear ineligible, and the in-between or unsure.

Title and Abstract Filtering

1. Was effect of bird perches, either natural or artificial, on any measure of seed dispersal or seedling establishment evaluated?
2. Did the study mention bird dispersers?
3. Was the study conducted in a degraded habitat near/adjacent a forest, regardless of type?
4. Is there full text available in English?

Full text filtering criteria

1. Was there a comparison of measurement between control (degraded area without perches) and treatment (degraded area with perch)?
2. Did the study reported data mostly contributed by birds, as justified by observations, previous literature, or pilot studies?
3. Did it provide raw data, descriptive and/or inferential statistics in Figure, Table, or text on at least one of the following: seed richness, seed density, seedling richness, and seedling density?

We will include studies that used a combination of perches and another attractor (i.e., food, water) in the review and consider their interacting effects in the meta-analysis, if possible.

### Study validity assessment

We will assess the quality of the studies accepted after full-text filtering based on risk of bias and method validity. We will use the CEE Critical Appraisal Tool [47] and assess the risk of bias using seven sub-criteria. As with most ecological studies, we expect a lot of studies to lack a blinding process during sample selection, intervention application, and/or outcome assessment, and hence contribute to the overall risk of bias assessment. We will use Criteria 3 for observational studies involving existing perches (e.g., tree islands) and Criteria 4 for experimental studies that modified or added perches (e.g., artificial posts). Lastly, we are interested in the raw data and/or descriptive statistics of the studies provided in the studies. But for studies where these are unreported, we will extract effect sizes from the inferential statistics and assess them under Criteria 7.

The method used to measure the effect of interest affects the reliability of the meta-analysis, hence we will adapt the second criteria to appraise method validity (Table 4). We assume that the best method for this kind of study (i.e., high validity) is the use of field-based observations of birds defecating seeds on the perches, because this allows us to establish for certain that the seeds or seedlings examined were from birds. However, we expect to find only a handful of studies with this ‘gold standard’. We also expect that the majority of studies will have employed seed rain collection as a method, which can produce varying data quality depending on the specificity of the sampling design. We note, however, that this method can be considered as high validity if it was designed to be bird-specific (Table 4).

**Table 4:** Study quality assessment based on external validity of methods

Method validity	High	Low
Methods to measure species richness and density of bird-dispersed seeds	Field observation of seed dispersal by birds on perches Seed rain collection representing dispersal by birds (i.e., daytime only to exclude bats, exclude non-bird dispersers through method)	Seed rain collection representing dispersal by animals and non-specific to birds (i.e., open at night, non-exclusion of other potential dispersers)
Methods to measure seedling species richness and density	Long-term monitoring of plots under perches with known seed rain data and with predator and herbivore exclusion set-up	Sampling under perch sites without known seed rain data

Both overall risk of bias and method validity will be included as an exploratory or sub-grouping variable in the meta-analysis to check the sensitivity of the results. We will assign each study an overall risk of bias score of low, medium, and high following the CEE tool guidelines. The results and conclusion will be presented with consideration to the risk of bias and limitations among the collated evidence. For consistency checking, all assessments will be cross-checked by a second reviewer, and disagreements will be discussed as a team.

### Data coding and extraction strategy

We will extract data from the accepted list of articles using a pre-designed datasheet with pre-coded options for a subset of the 34 columns to standardise data extraction for analyses (Table 5; Additional file 3). This data sheet has been pilot-tested with the 10 benchmarking articles. The study detail component of the data extraction will include details pertaining to the attributes of the study setting. The set-up fields refer to details on the methods and potential effect modifiers, namely matrix habitat type and distance of perch to forest. The outcome fields contain information about the data that are being compared. An open field (‘Notes’) allows to track unusual attributes associated with a specific study.

**Table 5:** Pre-tested data sheet for extracting data from the final list of accepted articles

Component	Label	Type	Description
Publication details	Study_no	Numerical	ID number
	Title	Free text	Title of the publication
	Lead_author	Free text	Name of the primary/first author
	Email	Free text	Email address of the primary/first author
	Pub_Year	Date	Year of publication
	Pub_type	Categorical	Type of publication
Study details	Year	Date	Year study was conducted
	Country	Categorical	Country where the study was conducted
	Lat	Numerical	Latitude in decimal degrees
	Long	Numerical	Longitude in decimal degrees
	Elevation	Numerical	Elevation in m.a.s.l
Set-up	Int_type	Categorical	Intervention type
	Height	Numerical	Perch height in meters
	Treesp	Free text	If intervention is isolated tree, indicate tree species
	Matrix	Categorical	Habitat type of the matrix
	Forest	Categorical	Type of forest
	Ctrl_size	Numerical	Number of samples for the control
	Int_size	Numerical	Number of samples for the intervention
	Distance	Numerical	Distance of perch to nearest forest in meters
	Length	Numerical	Length of experiment in days
	Method	Categorical	Method used in the study
Outcome	Out_type	Categorical	Type of outcome measured
	Out_spec	Free text	Notes on the specificity of outcome measured, whether the seeds or seedlings are native species, bird-dispersed, etc
	Unit	Free text	Unit of the mean values
	Int_mean	Numerical	Mean value of the intervention
	Ctrl_mean	Numerical	Mean value of the control
	Variance	Categorical	Type of variance measured
	Int_var	Numerical	Variance of the intervention
	Ctrl_var	Numerical	Variance of the control
	Stat_test	Free text	If mean and error not provided, input test statistics used for analysis and degrees of freedom
	Stat_value	Numerical	If mean and error not provided, input value of the statistical test used
	p-value	Numerical	If mean and error not provided, input probability measure used for hypothesis testing
	Other_var	Free text	Confounding variable that may affect the results
	Notes	Free text	General notes on reliability, data availability, limitations, assumptions

Categorical data fields have pre-coded options available

Data will be taken from the text, figures, and tables in the publication. Data reported in figures will be extracted using the metaDigitise package in R [48]. If the data is not readily available (i.e., incomplete parameters, transformed data), we will try to reach out to the corresponding author/s and ask for the missing data. As a back-up solution, missing data will be estimated through recalculation of statistics and data imputation methods, and these will be tagged via an accuracy column and checked for bias via sensitivity analysis [49]. The extracted data records, both raw and processed, will be uploaded in Figshare for data archiving and sharing and cited as part of the supplementary materials of the review.

We expect that some studies will contribute multiple effect sizes and steps will be taken to minimize effect size dependency. Results from studies that reported more than one outcome measure (e.g., seed richness and seedling density), collected data from several independent study sites, and used different perch types will be encoded separately with distinct effect sizes. Additionally, studies that specifically tested perches at varying distances from the forest will be considered as distinct results, if they have replicates, and stored as such in the data sheet. For example, a study that collected data from the control and perches at three different distances away from the forest (e.g., 5 m, 10 m, 15 m) will be treated as three distinct responses in the overall analyses. Some studies classified and reported seed rain data based on seed dispersal mode, type of plant (e.g., shrub, tree), and species residency status (e.g., native or non-native). For such cases, we will only use those specific to animal or bird dispersal, to seeds of forest tree species, and to native species, respectively, because these are the outcomes that are relevant to our question.

Two reviewers will independently extract data from all eligible studies, unless the number of studies exceeds 50. In that case, only one reviewer will extract the data and it will subsequently be cross-checked by a secondary reviewer. Discrepancies from the cross-checking will be discussed within the team for consensus building.

### Potential effect modifiers/reasons for heterogeneity

We will test for potential impacts of landscape context and environment on the relationship between perches and seed rain and seedling establishment. We will consider distance of perch to forest patch and type of matrix as landscape context specific effect modifiers, because of their role in facilitating or hampering movement of birds through the landscape [40, 50] and their relevance to addressing biodiversity threats from forest loss and fragmentation, globally [51]. We will also consider anomalies in annual rainfall and bioclimatic region as potential modifiers for the effect size estimates on seedling establishment outcomes because of the role of water availability in seed germination and survival [41].

The reported effect of distance to forest patch on vertebrate-dispersed seed rain in literature is mixed, with some reporting negative [52] or no effect [37]. Bird traits are important in this context [53]. In general, forest birds tend to stay within or near vegetated areas and are reluctant to venture into matrix habitats (i.e., degraded or open areas) due to increased risks from predation and reduced availability of resources [40]. Therefore, we expect that the probability of forest birds to cross the matrix is inversely associated with gap width (defined as distance from one patch to the nearest patch), and that the likelihood of birds to use perches is higher closer to forests overall. Matrix type in turn can affect movement decisions of birds by influencing the cost and benefit of movement steps when travelling between patches [54]. Matrix habitat comprising dense, tall vegetation for example, can provide cover from predators (lower cost) but can also limit visual perception of the landscape (higher cost). Whilst matrix habitat of similar structure to habitat associated with a specific bird species has been confirmed to allow for increased movement rates in a recent meta-analysis [40].

We will compile the rainfall data from the weather station located nearest to each study location and we will compute a long-term historic probability distribution of total annual rainfall for each site. We will subsequently identify whether the year of the study (or any of the preceding 2 years) fell within extreme wet, very wet, extreme dry, or very dry years, defined as those with total annual precipitation > 90th percentile of the historical distribution, 55th–90th, < 10th and 10th–55th percentile, respectively [55]. Average rainfall years will be defined as those where total annual precipitation is between the 45th and 55th percentile. We will also examine whether effects will vary among bioclimatic regions (i.e., tropical, subtropical, temperate, and boreal), which differ due to differences in geology, biodiversity, disturbance regimes, and land use history.

Lastly, we will assess to what extent the quality assessment scores (Table 4 and CEE Critical Appraisal Tool) will influence the effect size estimates.

### Data synthesis and presentation

We will provide a narrative synthesis and—if data allow—a quantitative synthesis (sensu meta-analysis) of the studies included in the final list. The narrative synthesis will report on the distribution of studies that have been conducted thus far and data availability. Based on the representation in the study details of the studies, we can identify sub-topics and research gaps that warrant further research. The meta-analysis is dependent on the number of studies with extractable data.

The effect size will be computed as the unbiased standardized mean difference Hedges’* g* [56]. Positive *g* values indicate higher seed richness, seed density, seedling richness, and seedling density in areas with perches than in those without perches, and vice versa. This metric has the following equation

$$g = \frac{{X^{E} - X^{C} }}{s} J,$$

representing the difference in the mean (*X)* of the experimental (E) and control group (C), standardized by the pooled standard deviation (s) and includes a correction factor (*J*) for small sample size [49] which are computed as

$$s = \sqrt {\frac{{\left( {n_{E} - 1} \right) s_{E}^{2} + \left( {n_{C} - 1} \right) s_{C}^{2} }}{{n_{E} + n_{C} - 2}}} ,$$

$$J = 1 - \frac{3}{{4 \left( {n_{E} + n_{C} - 2} \right) - 1}},$$

where $s_{E}$ and $s_{C}$ correspond to standard deviations, and $n_{E}$ and $n_{C}$ to sample sizes of the two groups. The effect size variance $(v_{d} )$ will be obtained, using

$$v_{d} = J^{2} \left( {\frac{{n_{E} + n_{C} }}{{n_{E} n_{C} }} + \frac{{g^{2} }}{{2 \left( {n_{E} + n_{C} } \right)}}} \right).$$

We will estimate the effect sizes for two seed-related outcomes (i.e., seed density and seed richness) and two seedling-related outcomes (i.e., seedling density and seedling richness). Both seed supply and seedling recruitment need to be improved for perches to be considered effective for ANR [57]. To handle effect size dependency, we will use a multi-level random-effects meta-analytical model approach with study identification as a random effect. Variance will be estimated using multiple estimators, namely DerSimonian-Laird, Paule-Mandel, Maximum Likelihood, Restricted Maximum Likelihood and Sidik-Jonkman estimators, as part of a sensitivity analysis to check how estimation methods may influence the results [58]. The distribution of the total variance across the levels will be examined, and the three-level model will be compared with null models, where the variance of one or both levels are held constant (i.e., within-study variance constrained, between-study variance constrained, and both level variance constrained), following the approach described in Assink and Wibbelink [59]. The fit of the four models will then be compared using ANOVA and evaluated using AIC values.

We will also assess the effect of potential modifiers through a mixed-effects meta-regression approach. We will fit the effect size estimates for seed-related outcomes to distance to forest and matrix type as fixed effects, and similarly for the seedling-related outcome but with the addition of precipitation variation. All computations and visualization of forest plots will be done in R using the metafor package [60].

Small study effects, including publication bias and time-lag bias, will be tested using multilevel meta-regression following Nakagawa et al. [61]. For this, we will fit meta-regression models with the square root of the inverse of effective sample size and publication year as moderators [61]. We selected this test given its performance in handling heterogenous and non-independent data, mainly to detect bias and secondarily to compute bias-corrected effect size estimates as part of a sensitivity analysis. We acknowledge that the ‘corrected’ estimates may still be overestimated or underestimated and these will be interpreted cautiously. The resulting systematic review and/or meta-analysis will be reported with the PRISMA-EcoEvo checklist [62].

## Supplementary Information

**Additional file 1.** Search string and database searches. List of benchmark articles, search string development, and databases included in the search.

**Additional file 2.** Eligibility criteria and pilot-testing. The eligibility criteria for title and abstract level and full-text screening level and the pilot-testing results on 20 articles.

**Additional file 3.** Data extraction sheet. The standard data sheet for data extraction and the pilot-tested data extracted from 10 benchmarking articles.

**Additional file 4.** Systematic review stakeholder survey. The questionnaire used for the stakeholder survey.

**Additional file 5.** ROSES for systematic review protocols. The ROSES checklist.

## Data Availability

All data generated or analysed from the pilot testing are included in this published article and its Additional files.
