# Supplementary material for: How effective are perches in promoting bird-mediated seed dispersal for natural forest regeneration? A systematic review protocol
Source: Environ Evid. 2023 Aug 3;12:15. doi: 10.1186/s13750-023-00308-z (PMC11378788; doi:10.1186/s13750-023-00308-z)
Supplement: Supplementary file 4 — Additional file 4. Systematic review stakeholder survey. The questionnaire used for the stakeholder survey. [file 13750_2023_308_MOESM4_ESM.docx]

Systematic Review Stakeholder Survey

INTRODUCTION:

The team plans to conduct a systematic review around the research question “How effective are perches in promoting bird-mediated in degraded landscapes for natural forest regeneration?”. We aim to assess whether perches, both natural and artificial, in the degraded areas near forests can help accelerate forest regeneration. From the results, we hope to recommend reforestation strategies based on the assisted natural regeneration (ANR) approach, which are said to be more cost-effective and more beneficial to biodiversity.

To do so, we will systematically search all relevant literature using a protocol. As stakeholders, we kindly ask for your feedback and insight about the topic and the search strategies. This will help minimize bias and increase transparency of the review process.

1. Respondent Information

Name:

Email:

Affiliation:

Position/Occupation:

Do you consider yourself as a stakeholder in the context of forest restoration? _______________

1. Topic exploration
2. Are there research questions or subtopics regarding the use of artificial and natural perches to promote seed dispersal and seedling establishment for forest regeneration that you believe require more research?
3. Based on your experience and knowledge, what habitat elements in the degraded areas do fruit-eating birds use as perches?
4. Aside from perches, what other ways can we attract birds to the degraded habitats to promote seed dispersal?
5. Search strategy
6. Are there other sources of information (databases, organizational websites) you suggest we include in our search beyond the ones listed below?
7. Listed below are the search terms we will use to find relevant literature. Are there any additional search terms you would use if you were conducting the literature search?

| **Elements** | **Search terms** |
| --- | --- |
| Population | Bird  Avian  Aves  disperse* |
| Artificial Perches | Perch*  artificial perch*  roost* |
| Natural Perches | fruit*  nucleation*  nuclei  tree isl*  woodland isl*  habitat isl*  remnant tree*  isolated tree*  single tree*  shrub* |
| Outcome | seed dispersal  seed rain*  seedling* |

* indicates searching for word variations (e.g., Perch* can be perches or perching)

1. Data privacy

Do you allow us to identify you as a stakeholder in the published systematic review?

___ Yes ___ No

May we share your responses in the published systematic review?

___ Yes ___ No ___ Please contact me first
